# Where’s the cookie? The ability of monkeys to track object transpositions

**DOI:** 10.1007/s10071-018-1195-x

**Published:** 2018-06-01

**Authors:** Katarzyna Majecka, Dariusz Pietraszewski

**Affiliations:** 1 Department of Experimental Zoology and Evolutionary Biology, Faculty of Biology and Environmental Protection, University of Lodz, Banacha 12/16, 90-237 Łódź, Poland; 2 Department of Ecology and Vertebrate Zoology, Faculty of Biology and Environmental Protection, University of Lodz, Banacha 12/16, 90-237 Łódź, Poland

**Keywords:** Object permanence, Transposition, Monkey, Ape, Dog, Piaget, Cognitive abilities

## Abstract

Object permanence is the ability to represent mentally an object and follow its position even when it has disappeared from view. According to Piaget’s 6-stage scale of the sensorimotor period of development, it seems that object permanence appears in Stage 4 and fully develops in Stage 6. In this study, we investigated the ability of some species of monkeys (i.e. pig-tailed macaque, lion-tailed macaque, Celebes crested macaque, barbary macaque, De Brazza’s monkey, L’Hoest’s monkey, Allen’s swamp monkey, black crested mangabeys, collared mangabeys, Geoffroy’s spider monkey) to track the displacement of an object, which consisted of a reward hidden under one of two cups. Our findings showed that the examined subjects possess Stage 6 of object permanence. We then compared our results with data on apes and dogs participating in Rooijakkers et al. (Anim Cogn 12:789–796, 2009) experiment, where the same method was applied. The monkeys examined by us performed significantly better than the dogs but worse than the apes. In our experiment, the monkeys performed above chance level in all variants, but it should be noted that we observed significant differences in the number of correct choices according to the level of a variant’s complexity.

## Introduction

Object permanence is defined as the ability to understand that objects continue to exist even when they have disappeared from view. In other words, if an object changes position, the subject is capable of mentally tracking the object’s possible movements. This ability is considered to be the fundamental skill of spatial cognition (Jaakkola 2014).

According to Piaget’s 6-stage scale of sensorimotor development, it is only in Stage 4 that searching for a hidden object starts (8–12 months in human infants). However, if these children see an object in its first location and then this is hidden in another place, then they will seek it in the former location. This response is named an A-not-B or a perseveration error (Piaget 1954). Children between 12 and 18 months are able to find an object when it is hidden in multiple locations within their view (Piagetian Stage 5), but still have difficulties to find an object that is invisibly displaced. The subject who reaches Stage 5 overcomes the perseveration error and takes into consideration successive displacements. The full understanding of object permanence develops in a human infant between the ages of 18 and 24 months (Piagetian Stage 6). Then, a child can solve sequential invisible displacements and reconstruct the movements of an unperceived object (de Blois et al. 1998). It is believed that reaching this stage marks a milestone in children’s development (Piaget 1954). Object permanence and the ability to track a displaced object seem to be very important for many animals because every day they have to remember the location of predators or resources such as food. Piaget’s scale is also used to determine the degree of development of cognitive abilities in non-human species. So far, the solving of displacement tasks has come under scrutiny [reviewed in Jaakkola (2014)] in such animals as cats [reviewed in Shreve and Udell (2015)], dogs (Fiset and LeBlanc 2007; Rooijakkers et al. 2009), dolphins (Jaakkola et al. 2010; Johnson et al. 2015), sea lions (Singer and Henderson 2015), dwarf goats (Nawroth et al. 2015), parrots (Pepperberg and Funk 1990), and corvids (Pollok et al. 2000). Likewise, several species of monkey, such as long-tailed macaques (Schloegl et al. 2013), rhesus macaques (Filion et al. 1996) and cotton top tamarins (Neiworth et al. 2003), have become the subject of similar studies. Finally, great and lesser apes have also been examined (e.g. de Blois et al. 1998; Call 2003; Collier-Baker et al. 2006; Rooijakkers et al. 2009; Anderson 2012).

Comparisons of the ability of object tracking have been done in both non-human primates and children. Such comparisons for infants and great apes have been carried out under varying degrees of difficulty (e.g. invisible and visible displacement). Findings have shown that children 19 months, 2 years and 2.5 years old as well as great apes have very similar cognitive skills for dealing with the physical world (Call 2001; Barth and Call 2006; Collier-Baker and Suddendorf 2006; Herrmann et al. 2007).

According to Jaakkola’s (2014) classification, there are three experimental methodologies to be applied for understanding invisible displacement in non-human animals, namely, a Piagetian task, rotation and transposition. In a standard Piagetian task, the target object is hidden inside the displacement device, which is subsequently placed under one of three opaque containers. Next, the displacement device is removed and the experimenter shows the subject the empty device and then the subject starts to search for the object. In a rotation task, opaque containers are placed on a turntable. The experimenter places the target object under one of the opaque containers and rotates the turntable (90° or 180°). The third type of task, transposition, requires that the target object be placed directly in containers without the intermediate displacement device. Subsequently, one or two containers out of the two or three provided are moved. The way the cups are moved affects the level of difficulty of the task. If the containers cross path or the empty container moves into the initial position of the baited container, then it causes more difficulties for the subject to choose baited containers (Doré et al. 1996; Fiset and Plourde 2013; Jaakkola 2014; Rooijakkers et al. 2009). In all three tasks (i.e. Piagetian, rotation, transposition), the subject possesses a mental representation of the situation and points by logical inference to the correct position of the target object invisibly moved (Jaakkola 2014).

To claim that a species understands invisible displacement, three factors must be controlled for or ruled out: sensory cues, social cues and associative learning. If these factors are not controlled for, then they could affect the results of experiments. Thanks to them, the animal could complete the task successfully without conceptual understanding of invisible displacement (Jaakkola 2014). Sensory cues could be, e.g., smell in case of dogs or the ability to echolocate (cf. dolphins, bats). Social cues include unintentional body language or gaze of the experimenter. Associative learning might cause the animal to pass the test because they have learned simple associative rules such as ‘pick the location that the experimenter indicated’. Although the Piagetian framework may have some limitations, it is still very useful in examining and comparing cognitive abilities of human and non-human animals (Pepperberg 2002).

According to Amici et al. (2010), there is no overall clear-cut distinction in cognitive skills between apes and monkeys, and existing differences have often been overestimated (Tomasello and Call 1997). However, due to miscellaneous procedures applied in different experiments, it is hard to make comparisons between species. For this reason, we decided to apply the methods used by Rooijakkers et al. (2009), who compared dogs and great apes in experiments on their ability to track visually object transposition.

The purpose of this study was to investigate the ability of some species of monkeys to track the displacement of an object and to compare the results from the literature on apes and dogs, to which the same method was applied using the transposition task (Rooijakkers et al. 2009). In this study, we were also interested in evaluating how the subjects solve problems depending on the level of a variant’s complexity.

## Methods

### Ethical note

The experiment was non-invasive, the subjects participated in it voluntarily and they were neither food nor water deprived for the testing. The study received the approval of The Local Ethics Committee for Animal Experimentation (permit number 3/ŁB11/2016 of 18th January 2016) and acted in accordance with the law from 15th January 2015 on the protection of animals used for scientific purposes.

### Subjects

19 monkeys (7 females and 12 males), born in captivity, participated in the experiment (Table 1). The monkeys were housed at zoos across Poland: in Łódź (six individuals), Warszawa (one individual), Wrocław (nine individuals) and Poznań (three individuals). All individuals lived in indoor and outdoor enclosures, were fed their species-typical diet (vegetables, fruit, insects) and water was available at all times. For all subjects, the experiment involved rewards hidden inside cups and transposition tasks were a new experience.

**Table 1:** Monkeys included in the experiment

Subject (name)	Species	Sex	Age (years)	ZOO
*Cercopithecidae* Old World Monkey				
Grześ	Pig-tailed macaque *Macaca nemestrina* (Linnaeus, 1766)	Male	23	ZOO Łódź
Naomi	Lion-tailed macaque *Macaca silenus* (Linnaeus, 1758)	Female	11	ZOO Łódź
Rani	Lion-tailed macaque *Macaca silenus* (Linnaeus, 1758)	Female	21	ZOO Łódź
Woolfie	Lion-tailed macaque *Macaca silenus* (Linnaeus, 1758)	Male	26	ZOO Łódź
Punio	Lion-tailed macaque *Macaca silenus* (Linnaeus, 1758)	Male	13	ZOO Łódź
Taro	Celebes crested macaque *Macaca nigra* (Desmarest, 1822)	Male	17	ZOO Wrocław
Tyson	Barbary macaque *Macaca sylvanus* (Linnaeus, 1758)	Male	11	ZOO Wrocław
Lisbeth	Barbary macaque *Macaca sylvanus* (Linnaeus, 1758)	Female	2	ZOO Wrocław
Ries	Barbary macaque *Macaca sylvanus* (Linnaeus, 1758)	Male	11	ZOO Wrocław
Canail	Barbary macaque *Macaca sylvanus* (Linnaeus, 1758)	Male	9	ZOO Wrocław
Hiszpan	De Brazza’s monkey *Cercopithecus neglectus* Schlegel, 1876	Male	16	ZOO Łódź
Holly	L’Hoest’s monkey *Cercopithecus lhoesti* Sclater, 1899	Female	8	ZOO Wrocław
Pyza	Allen’s swamp monkey *Allenopithecus nigroviridis* (Pocock, 1907)	Female	19	ZOO Warszawa
Corso	Black crested mangabey *Lophocebus aterrimus* (Oudemans, 1890)	Male	19	ZOO Wrocław
Alf	Collared mangabey *Cercocebus torquatus* (Kerr, 1792)	Male	16	ZOO Wrocław
Olaf	Collared mangabey *Cercocebus torquatus* (Kerr, 1792)	Male	5	ZOO Wrocław
*Atelidae* New World Monkey				
Zosia	Geoffroy’s spider monkey *Ateles geoffroyi* Kuhl, 1820	Female	18	ZOO Poznań
Zuza	Geoffroy’s spider monkey *Ateles geoffroyi* Kuhl, 1820	Female	18	ZOO Poznań
Colombo	Geoffroy’s spider monkey *Ateles geoffroyi* Kuhl, 1820	Male	17	ZOO Poznań

### Warm-up

The monkeys were tested in the indoor or outdoor enclosures in which they lived. Enclosure selection depended on factors such as good visibility of the platform by the subject, not finding any trace of the subject’s anxiety, and weather conditions. A wooden platform (60 × 30 cm) was attached outside of a mesh panel. There were four small dots on the platform at a distance of 15 cm from each other indicating the location of the cups. Two identical opaque grey cardboard cups (diameter 8 cm, 9 cm high) were used in the experiment. During the experiment, fruit and insects (species-typical diet) were provided as a reward.

All subjects underwent a warm-up phase prior to the experiment with the aim to familiarise them with the procedure of disappearance of food under cups and receiving it after touching the cup. The warm-up phase took place at the same location where the experiment was later carried out using the same platform and cups. The platform was fixed to the net and left for 1 h to give the subjects time to get used to it. The warm-up phase was conducted by the experimenter (E1). During this phase, the cups were randomly distributed over the whole surface of the platform, but never in the locations where they were to be presented during the experiment. E1 showed the empty cups to the subject and then covered the reward with one of the cups. The individual received the reward after choosing the correct cup. In the situation when the subject pointed to the un-baited cup, E1 showed an empty cup and where the reward is, but subject did not get it. The warm-up phase was completed when the subjects touched one of the two cups (baited or un-baited) five times. At this stage, the experimenter did not manipulate the cups in the same way as during the actual part of the experiment.

### Procedure

**Fig. 1:**
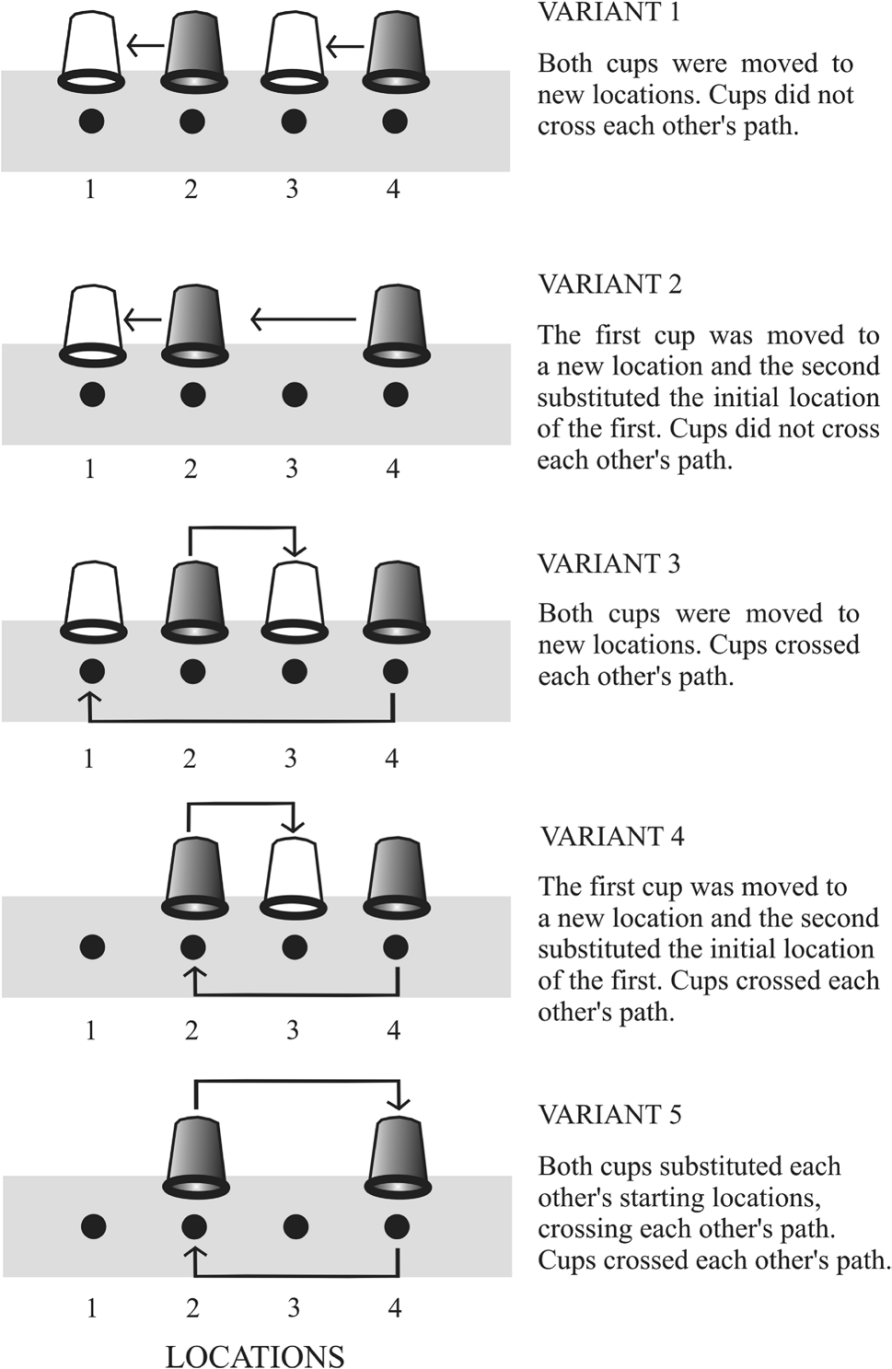
Schematic representation of the five different transposition variants performed in this study (according to Rooijakkers et al. 2009). The grey cups represent the initial position, while the white ones indicate the final position in given variants if the cups were relocated. In all variants the start locations were the same. The order of the variants presented by E1’s point of view: 2R, 5L, 1L, 3R, 4L, 5R, 1R, 3L, 4R, 2L, 2R, 5R, 1L, 3R, 4L, 5L, 1R, 3L, 4R, 2L (L, reward under left–hand cup; R, reward under right-hand cup)

The experiment always started in the same way. The forward-facing experimenter (E1), invariably the same person, sat in front of the subject, separated by the mesh panel and the platform, and obtained its attention by showing the reward. Once the subject focused on the task, E1 put the food reward on the platform (location 2 or 4; Fig. 1) and next showed two empty cups and then covered the reward with one of them. The initial position of the cups in all variants was always the same (location 2 and 4). After hiding the reward, the experimenter instantly proceeded to manipulate the cups. E1 simultaneously moved the cups from initial to final positions in the following way: the right-hand side cup with his right-hand and the left-hand side cup with his left hand. After the cups were put in their final positions (Fig. 1), the subject could touch either of them. It was only when the subject had made the right choice that it received the hidden reward. The second experimenter (E2), facing downwards, was present during all trials, read out the subsequent numbers of the variants and noted whether the selection made by the subject was correct or not. All the subjects underwent the experiment in the presence of five variants, each of them applied four times, summing up to 20 trials total. Determined by drawing lots at the outset of the experiment, the order of performing the random sequence of variants remained the same for each subject. The conditions of drawing lots stipulated that in each of the five task variants the food should be hidden twice under the right-hand side cup and twice under the left-hand side one. What is more, during one session, none of the variants could be repeated more than twice in a row. The variants were implemented as follows:

- *Variant 1* E1 grabbed the right cup with the right hand and the left cup with the left hand, then with one continuous movement he moved the cups so that the cup from location 2 would be placed on location 1, and the cup from location 4 on location 3.
- *Variant 2* E1 grabbed the right cup with the right hand and the left cup with the left hand, then with one continuous movement he moved the cups so that the cup from location 2 would be found on location 1, and the cup from location 4 on location 2.
- *Variant 3* E1 grabbed the right cup with the right hand and the left cup with the left hand. Then with one continuous motion, so that the arms would cross over the platform, moved the cup from location 2 to location 3, and the cup from location 4 to location 1.
- *Variant 4* E1 grabbed the right cup with the right hand and the left cup with the left hand. Then with one continuous motion, so that the arms would cross over the platform, moved the cup from location 2 to location 3, and the cup from location 4 to location 2.
- *Variant 5* E1 grabbed the right cup with the right hand and the left cup with the left hand. Then with one continuous motion, so that the arms would cross over the platform, moved the cup from location 2 to location 4, and the cup from location 4 to location 2 (Fig. 1).

After completion of the experiments, a control test was performed in which the experimenter (E1) covered the cups while placing the food to prevent the subject from knowing where it was hidden. The control procedure resembled the experiment, but the monkey received the reward regardless of the cup choice. The control test aimed at the exclusion of such factors as the use of scent or unconscious hints given by E1.

The subjects entered the experiment voluntarily and could stop the experiment at any moment, departing from the place where it was carried out. Some subjects completed the experiment during one session, whereas some subjects lost interest in working with the experimenter. The experiment was continued when the subject again approached the place of the experiment.

### Data scoring and analysis

Differences between variants were analysed by the ANOVA Friedman test. In case of significant effects, a post hoc test (Conover-Iman) was conducted. Second, the Wilcoxon test was used to determine whether the results of the experiment in each variant were above chance. Third, differences (calculated separately for each variant) were investigated between the results of the experiment and the control test using a non-parametric exact two-tailed statistic (Wilcoxon test). Finally, the findings obtained from the studied monkeys were compared with the raw scores of dogs and apes as available from the literature (Rooijakkers et al. 2009). To determine differences between dogs (*N* = 20) and monkeys (*N* = 19), and between apes (*N* = 8) and monkeys, the Mann–Whitney *U* test was performed separately for each variant.

## Results

The individual results of the subjects tested in each variant are shown in Table 2.

**Table 2:** Sum of correct choices of each subject per variant (with four trials in each variant)

Subjects	Species	Variant				
		1	2	3	4	5
Grześ	Pig-tailed macaque	3	3	3	2	4
Naomi	Lion-tailed macaque	4	4	4	4	4
Rani	Lion-tailed macaque	4	3	2	2	3
Woolfie	Lion-tailed macaque	3	3	3	4	3
Punio	Lion-tailed macaque	4	3	3	2	1
Taro	Celebes crested macaque	3	3	3	3	4
Tyson	Barbary macaque	3	4	3	4	3
Canail	Barbary macaque	4	4	4	4	3
Ries	Barbary macaque	4	3	4	4	4
Lisbeth	Barbary macaque	4	3	2	2	3
Hiszpan	De Brazza’s monkey	4	4	4	1	3
Holly	L’Hoest’s monkey	4	4	4	2	2
Pyza	Allen’s swamp monkey	4	3	2	2	2
Corso	Black crested mangabey	4	3	4	4	3
Alf	Collared mangabey	4	4	4	4	3
Olaf	Collared mangabey	4	4	3	4	4
Zosia	Geoffroy’s spider monkey	4	4	3	2	2
Zuza	Geoffroy’s spider monkey	4	4	3	2	3
Colombo	Geoffroy’s spider monkey	3	3	3	2	3

### Differences between variants

**Fig. 2:**
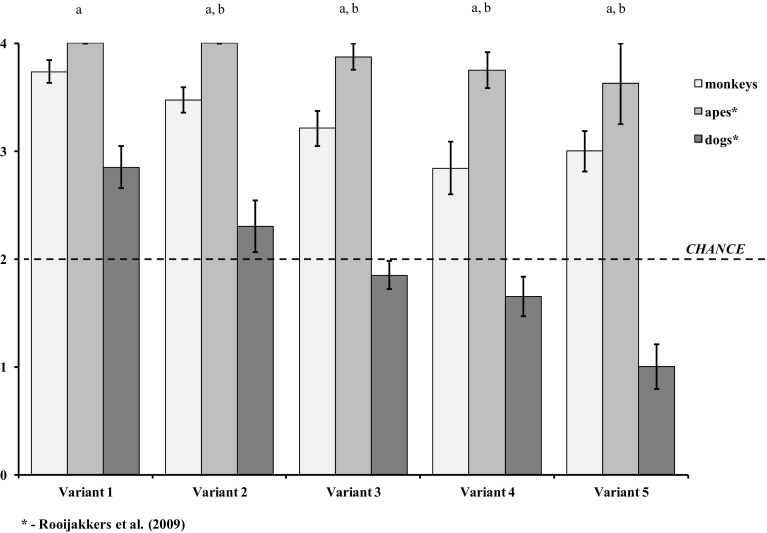
Mean number (± SEM) of correct response of the monkeys (*N* = 19) in the current study and the dogs (*N* = 20) and the apes (*N* = 8) from Rooijakkers et al. (2009) for each of the five variants. ^a^Statistically significant differences between the dogs and the monkeys (*P* < 0.01). ^b^statistically significant differences between the monkeys and the apes (*P* < 0.05)

A significant effect of variant was observed (ANOVA Friedman test *χ*^2^ = 13.40, *n* = 19, *df* = 4, *P* < 0.01). Post hoc Conover-Iman test showed that monkeys performed better in Variant 1, where both cups were moved to new locations without crossing each other’s path, compared to Variants 3, 4 and 5, where cups crossed each other’s path. The subjects also achieved better results in Variant 2, where one cup moved to a new location and then substituted the initial location of the first without crossing each other’s path, compared to Variant 4 (Table 3). Overall, the examined monkeys performed above chance in all variants (Wilcoxon test *z* > 2.65, *P* < 0.01 in all variants) (Fig. 2). When treated as a group, the subjects showed significant differences between the performance of the experiment to the result of the control test in each variant, which excludes other external factors affecting selection of the cup during the test (Table 4).

**Table 3:** Results of post hoc Conover-Iman test comparing results of examined monkeys depends on variant of experiment (*P* value)

	Variant 1	Variant 2	Variant 3	Variant 4	Variant 5
Variant 1					
Variant 2	0.285				
Variant 3	**0.021**	0.205			
Variant 4	**0.002**	**0.030**	0.349		
Variant 5	**0.003**	0.055	0.503	0.789	

Significant values are highlighted in bold

**Table 4:** Wilcoxon test results comparing the performance of the subjects to the result of the control test

	*T*	*z*	*P*
Variant 1	0	3.62	< 0.001
Variant 2	0	3.62	< 0.001
Variant 3	9	3.05	0.01
Variant 4	5	2.67	0.01
Variant 5	15	2.13	0.03

### Comparison of results with dogs and apes

The monkeys subjected to the experiment achieved significantly better results than dogs (as reported in Rooijakkers et al. 2009) in all five variants (*Z* > − 4.83, *P* ≤ 0.001 in all cases Table 5; Fig. 2). However, the monkeys scored worse than apes (Rooijakkers et al. 2009) in Variant 2, 3, 4 and 5. It was only in Variant 1 that no statistically significant differences were noted (Table 5; Fig. 2).

**Table 5:** Comparison of the results of the examined monkeys and dogs and the results of monkeys and apes [data on dogs and apes from Rooijakkers et al. (2009)] (Mann–Whitney *U* test)

Comparison	Monkeys	Apes	Dogs	Monkeys/dogs		Monkeys/apes	
	Mean (± SD)	Mean (± SD)	Mean (± SD)	*Z*	*P*	*Z*	*P*
Variant 1	3.74 (± 0.45)	4.00 (± 0.00)	2.85 (± 0.88)	− 3.23	0.001	1.54	0.12
Variant 2	3.47 (± 0.51)	4.00 (± 0.00)	2.30 (± 1.08)	− 3.57	< 0.001	2.51	0.01
Variant 3	3.21 (± 0.71)	3.88 (± 0.35)	1.85 (± 0.59)	− 4.59	< 0.001	2.33	0.02
Variant 4	2.84 (± 1.07)	3.75 (± 0.46)	1.65 (± 0.81)	− 3.21	0.001	2.02	0.04
Variant 5	3.00 (± 0.82)	3.63 (± 1.06)	1.00 (± 0.92)	− 4.83	< 0.001	2.26	0.02

## Discussion

In the present study using the transposition task, monkeys were successful in all variants, both in the case of a simple displacement of the two cups and in displacements with reverse swapping. However, there were differences in the number of correct choices according to the level of a variant’s complexity. Generally, it was easier for the subjects to track the position and find the hidden reward if the cups did not cross each other’s path during movements. In fact, the monkeys obtained worse results if the paths of the two cups were crossed and the two containers exchanged locations with one another. Although monkeys performed above chance level in all variants, they scored significantly worse than the apes examined by Rooijakkers et al. (2009), except for Variant 1. In the latter experiment, which employed exactly the same procedure of this study, chimpanzees, bonobos, orangutans and gorillas succeeded in all variants, and the level of difficulty did not affect the results of the apes (Rooijakkers et al. 2009). In another experiment using a Piagetian task, which involved object transposition in the presence of the above-mentioned ape species, all subjects selected the correct cup above chance level and there were no significant differences amongst single and double (the reward changed the position twice) displacement tasks (Barth and Call 2006). Call (2001) observed that orangutans, chimpanzees and 26-month-old children performed above chance level on visible and invisible displacement tasks, except for nonadjacent invisible displacements (i.e. the reward was moved to a nonadjacent container). In another of Call’s (2003) experiments, several tasks were presented to chimpanzees and orangutans. In the no-landmark transposition (food is presented directly to the subject), apes performed better than in the landmark (the subject had to infer the position of the reward using a landmark) transposition test, whereas in the latter subjects obtained significantly better results in the no-swap transposition compared to the reverse swap.

Overall, the results obtained by monkeys vary among experiments using a Piagetian task. For instance, long-tailed macaques performed above chance level in single and double transpositions (Amici et al. 2010), spider monkeys only in a single transposition, but neither coped with a reverse transposition, that is, when the baited cup and empty cup switched location twice and returned to their original position. Experiments on cotton top tamarins revealed that their performance was above chance level in both visible and invisible displacement tasks and only in one (i.e. double invisible displacement with the second cup manipulated by the experimenter) did results dropped to chance level (Neiworth et al. 2003). Even though the procedures adopted in the aforementioned experiments differed, it is certain that a transposition which comprises crossing paths poses difficulty to many species of primates. It is solely apes that tackled such tasks successfully regardless of difficulty level.

The monkeys used in the present experiment scored significantly better in all tasks than the dogs examined by Rooijakkers et al. (2009), which performed above chance only in Condition 1 (i.e. both containers moved to a new location and not crossing each other’s path). If the containers changed their position in such a way that they crossed each other’s path, then the dogs could not follow the transition of the reward. This seems to indicate that monkeys exhibit more flexibility in mental representation compared to dogs. Rooijakkers et al. (2009) assumed that great apes use their mental representation more flexibly than dogs. Undoubtedly, one must also concur with Rooijakkers et al. (2009), who noted that dog inferential abilities are better expressed in the olfactory than in the visual modality. Moreover, differences in cognitive abilities between dogs and primates are well illustrated by Bräuer et al.’s (2006) research. Their experiments showed that dogs perform better at finding hidden food using communicative cues given by the human experimenter, whereas apes do better when the reward causes a noticeable change in the physical world (e.g. by generating a noise). Generally, the cup manipulated by the experimenter was preferred by the examined dogs and they achieved almost 50% better results than the apes in understanding communicative cues (Bräuer et al. 2006). The latter experiment provided arguments for apes’ developed ability to make inferences about the working of the physical and social world. In nature, apes are forced to gain food from hidden locations (e.g. underground nests), which requires causal understanding, and sometimes they need to use a tool. Probably because of this, they have evolved to develop skills in reading causal cues (Bräuer et al. 2006).

Dogs are considered to have an ability to read human behaviour and use triadic communication, i.e. one individual informs another about the location of various things, including food resources, for example, by pointing or other gestures. Their skills in understanding cooperative signs have developed over thousands of years of domestication (Coppinger and Coppinger 2002; Hare and Tomasello 2005; Bräuer et al. 2006). On the other hand, as a result of domestication, dogs could have lost some skills necessary for causal understanding due to lack of subsistence problems thanks to humans (Bräuer et al. 2006; Rooijakkers et al. 2009; Wynne 2016). Furthermore, ontogenetic development of interspecies cooperative skills in a dog may well result from the relationship established with a human in the first weeks of its life, which is usually not the case with primates subjected to experiments (Miklósi et al. 2003; Wynne et al. 2008).

According to the hypothesis of Natale et al. (1986), monkeys’ progress is limited to Stage 5 on the Piagetian scale of sensorimotor development of object permanence. The authors of that study presumed that only apes, hence not monkeys, could solve a task with the use of mental representation. However, the results of the present experiments as well as those of other researchers have given evidence for the reaching of Stage 6 by the examined monkeys. The species of monkeys that demonstrated good cognitive abilities is the cotton top tamarin, and results have shown that they possess Stage 6 object permanence (Neiworth et al. 2003). On the other hand, another monkey species, namely, the squirrel monkey, did not possess this stage (de Blois et al. 1998). Amongst primates, it was only lemurs that demonstrated Stage 5 of object permanence, as they failed in invisible displacement tasks—a result explained by Deppe et al. (2009) to be of ecological relevance. According to their hypothesis, lemurs’ ability to obtain stationary fruit or leaves and to avoid predators such as raptors, snakes and viverrids does not have to go beyond solving a visible displacement task. Lemurs are attacked by fossas, a large cat-like carnivore in Madagascar, only during sleep. Snakes hunt them using sit-and-wait tactics, whereas raptors observed through foliage can appear in and disappear from the field of view (Goodman 2003). On the other hand, in animals which are at risk of attack by terrestrial stalking predators such as felines, possessing Stage 6 object permanence is very important (Deppe et al. 2009).

In the case of non-primate animals, some species of birds can achieve a high level of cognitive abilities. Four species of parrots (i.e. an African Grey parrot, an Illiger macaw, a cockatiel, and a parakeet) showed Stage 6 competence (Pepperberg and Funk 1990), and this level was also achieved by Eurasian jays (Zucca et al. 2007). Zucca et al. (2007) concluded that a Stage 5 competence would suffice for those birds to collect food, even though it is not enough for protecting their caches of food. Successful protection of food is probably associated with the understanding of intentions and the manipulation of potential pilferers (Clayton and Dickinson 1998).

The human brain was designed by natural selection to solve adaptive problems faced by our hunter–gatherer ancestors (Duchaine et al. 2001). Obviously, the same process can be observed by examining the brain and adaptations in non-human species. The importance of cross-species comparisons to test evolutionary functions of organismic design is highly emphasised by evolutionary biologists, comparative psychologists, and behavioural ecologists. In this respect, an analysis of results regarding cognitive traits showed that they probably evolved independently amongst several vertebrate groups including Primates (Reader et al. 2011; van Horik et al. 2012). The results of the examined species of monkeys, as well as data from the literature on corvids, parrots, cetaceans, and apes, showed that the understanding of object permanence in such species is at a similar stage of development (Stage 6 competence). According to van Horik et al.’s (2012) hypothesis, cognitive traits might have arisen in distantly related groups (e.g. corvids and apes) because they have evolved to solve similar environmental problems. Having analysed many biological, ecological, behavioural and social system traits, the authors of that study conclude that, despite having different brain structures, there are striking similarities in the diet, use of tools, and social systems in corvids, parrots, apes, cetaceans, and elephants. Van Horik et al.’s (2012) study also speculated that species characterised by a relatively large brain and that undergo a long developmental period, that live a long life and live in a fluctuating habitat, could all be considered candidates for convergent evolution of cognitive abilities. It is, therefore, believed that the monkeys studied in the present experiment match well with the aforementioned groups of vertebrates. Finally, it is worthwhile to investigate further the cognitive abilities of other, hitherto not yet examined species, and particularly monkeys, because some major advances in our understanding of evolution have resulted from a convergence of data from numerous species (Sell 2012).
